# Supplementary material for: “We will need to build up the atmosphere of trust again”: Service providers’ perceptions of experiences of COVID-19 amongst resettled refugee adolescents
Source: PLoS One. 2023 Mar 24;18(3):e0283599. doi: 10.1371/journal.pone.0283599 (PMC10038302; doi:10.1371/journal.pone.0283599)
Supplement: Attachment — Submitted filename: plos one perception-COVID-10.docx [file pone.0283599.s001.docx]

To Author(s).

Date 28/04/2022

**Reviewer comments**

**Dear /sir, I hope this message find you well. These are Constructive Comments forwarded for the Author(s).i will appreciate for your concern if you address the given comments, questions or suggestion on time.**

The topic is very interesting which shows the impact COVID-19 on the refuge settlements that helps the issues to contribute for the tackling of the pandemic of COVID-19. In order to make your manuscript to the standards of scientific community in sharing knowledge and experiences of your work with other scientific or researchers the following points are forwarded. Overall Check for grammars/spellings error carefully. **:** re writes and make the title short beside this Make: COVID-19 impact on educational and support services for resettled adolescent refugee

Make keywords: less or = 5, use plos one guide line while writing the abstract…generally.

Avoid; the use of”we” ‘our’ …rather use the researcher /investigators/author[s].

Under the methods explain the benefit, disadvantage , and reasons for choosing content analysis in relation to your study.

we aimed: to describe the perspective of educators and other school-affiliated service providers on the impact of the COVID-19 pandemic on mental health and wellbeing of adolescent resettled refugees; to examine educators’ and other service providers’ views on the impact of the COVID-19 pandemic on access to and quality of education and support services for adolescent resettled refugees; and to explore adaptations and innovations in service delivery in the context of the COVID-19 pandemic

Please make the objective very **concise, and brief, that means make the general objective broad rather than breaking in to pieces.**

**In writing Abstract state the elements /components to be included like introduction/background, objective, methods, results…conclusion.**

**Selects terms while writing’** Our findings’ better to use the current study finding , this study , the finding of this study….

Give space after every paragraph e.g. Pp: 13 and discussion and conclusion part need space over all.

please upload the revised Manuscript in the form of (DOC, DOCX), Regarding references I hope you did it that was nice. Put cation for accordingly, too in the text where to be cited

**Old reference >>>recent one**

Hsieh H-F, Shannon SE. Three Approaches to Qualitative Content Analysis. Qual Health Res. 2005 Nov 1;15(9):1277–88.

Sinclair MF, Christenson SL, Lehr CA, Anderson AR. Facilitating Student Engagement: Lessons Learned from Check & Connect Longitudinal Studies. Calif Sch Psychol. 2003;8:29–41.

Good luck

Thanks for your response

JA Ahmed
