# Supplementary material for: “We will need to build up the atmosphere of trust again”: Service providers’ perceptions of experiences of COVID-19 amongst resettled refugee adolescents
Source: PLoS One. 2023 Mar 24;18(3):e0283599. doi: 10.1371/journal.pone.0283599 (PMC10038302; doi:10.1371/journal.pone.0283599)
Supplement: Attachment — Submitted filename: Salama covid reviewer table_SM[40].docx [file pone.0283599.s002.docx]

**Title:** “We will need to build up the atmosphere of trust again”: Perceptions of COVID-19 impacts on resettled refugee adolescents and educational and support services in the United States

**Manuscript ID:** PONE-D-22-05430R1
**Journal:** PLOS ONE

|  | **Reviewer comment** | **Response to reviewer** | ***Revised text*** |
| --- | --- | --- | --- |
| **Reviewer 1** | | | |
| 1 | The authors have addressed my concerns. The discussion is much clearer now. I have two thoughts that do not need to be addressed but that I wonder about; the data were collected at different points in the pandemic and I wondered if the themes differed based on when the data were collected. However, I think that they were also collected in different locations and under different circumstances as well so this may not be an answerable question. | This is an interesting point. As the  reviewer indicates, there are a  number of other factors such as  location that impacted  on the themes. In the  course of some interviews, it was  clear that respondents were experiencing different phases of school closures and reopenings, however, we did not expand on this point as we cannot fully determine how or if phase of the pandemic impacted the themes. |  |
| 2 | The other observation that repents felt that some students found working from home less distracting and I wondered if, for some students, the school is not a safe space and so attending remotely may have been a safer option. | This is possible but could not be evidenced by our data. |  |
| 3 | I don't need or expect a response to either of these points....just musing....a very interesting paper! | Thank you for these interesting questions and your helpful feedback. |  |
| **Reviewer 2** | | | |
| 1 | 1. Trustworthiness of the data analysis should be demonstrated. | We have added text indicating the methods we utilized to establish trustworthiness in thematic analysis. | “The research team drew on Nowell et al.’s guidance regarding establishing trustworthiness through the process of thematic analysis in several ways. For example, at the phase of data familiarization, the research team read and re-read interview transcripts, taking notes on potential codes, themes and interconnections. In the phase of generating initial codes, the research team kept a careful audit trail of all potential codes and codes that had been combined or deleted. In the phase of searching for themes, the team met and discussed how particular codes combined into larger themes, and all data analysis team members reviewed and approved the final coding and thematic scheme.” |
| 2 | 2. In the data analysis: As the directed content analysis was used, the pre-existing theory should be described up front clearly. | Directed content analysis can also be used based on prior research/ findings, rather than specifically based on pre-existing theory. In the case of our data analysis, we utilized directed content analysis due to the prior research during the SALaMA study, rather than a specific theory. |  |
| 3 | 3. In the discussion section: the authors should state if their findings offer supporting or non-supporting evidence for the pre-existing theory. In addition, any newly identified categories should be discussed in terms of whether they contradict or enrich the original theory. | As per our response above, we do not employ a specific theory in this paper, but rather draw on previous research to direct our thematic analysis. |  |
| 4 | 4. The authors aim to demonstrate the impact of the COVID-19 pandemic on the mental health and wellbeing of adolescent resettled refugees; however, the data does not fully support this conclusion. | We have reworded the objective of the study to respond to the reviewer’s comment. We agree with the reviewer that the findings reflect service providers' perspectives only and that we have been very careful to avoid claiming otherwise throughout the article. However, we agree that this could be better expressed in the title and so we have changed the title to “”We will need to build up the atmosphere of trust again”: Service providers’ perceptions  of experiences of COVID-19 impacts amongst resettled refugee adolescents.” | “The objective of this analysis, based on two phases of qualitative data collection for SALaMA, is to describe the perspective of educators and other school-affiliated service providers regarding experiences of the COVID-19 pandemic on mental health and wellbeing amongst adolescent resettled refugees and access to and quality of education and support services for adolescent resettled refugees.” |
| 5 | 5. The data collection process was multiphase and part of another study [SALaMA]. For the readers to understand how the process evolved, it would be better if a flow chart was used to display the research journey clearly and concisely. |  |  |
| 6 | 6. The title is misleading and does not accurately say what the study is about. It is better to use a more direct and clear title. | We agree with the reviewer that the findings reflect service providers' perspectives only and that we have been very careful to avoid claiming otherwise throughout the article. However, we agree that this could be better expressed in the title and so we have changed the title to:  “We will need to build up the atmosphere of trust again”: Service providers’ perceptions of experiences of COVID-19 amongst resettled refugee adolescents” |  |
| **Reviewer 3** | | | |
| 1 | 1-Before analysis the data by using the Dedoose qualitative software, the authors should try-out in other group that is similar the sample group in this study. That garantee the data collection of this study is suitabale for using the Dedoose qualitative software. | We were careful to check that Dedoose was the appropriate program for the purposes of our study. According to the Dedoose website, “Dedoose is a web-based application that allows you to organize and analyze research data, no matter what form those data take….Dedoose’s design is flexible enough for you and your team to apply whatever methodologies you choose. Dedoose supports both qualitative approaches and mixed methods approaches to data interaction and analysis in a secure, collaborative environment.” As such, we are confident that Dedoose is an appropriate software package for our purposes. |  |
| 2 | 2-In the result part of this study the authors might summary the relationship between variables of this study. | As this is a qualitative study, we do not categorise the results into variables. Summary of the relationship between variables would be more appropriate in the case of a quantitative analysis. |  |
| **Reviewer 4** | | | |
|  | Overall, this article is important, interesting, and useful. However, it should be improved a bit as follows. |  |  |
|  | The Abstract should be written in one paragraph, not divided into sub-headings. | Thank you, we have removed the sub-headings in the abstract. |  |
|  | Research articles from other parts of the world may also be included in the discussion. And these research articles should be considered for reference. --> Urairak, B. (2022). Effect of the COVID-19 Pandemic Related Mental Health on State Anxiety in Thailand. Asian Administration and Management Review, 5( 1), 1-6. --> Mustari, N., Herman, Aris, M., Mawardi, A., & Chaminra, T. (2021). The Effect of Online Learning Policy in the Era of Covid-19 on Students’ Quality. Asian Political Science Review, 5(2), 1-8. | We agree that research from other parts of the world is useful to integrate into the discussion section. Given the specifics of the study population, we have searched for literature and evidence that directly relates to resettled refugees – either, different populations of resettled refugees in the US or other resettlement contexts (Canada or Australia). | These findings are also echoed in research on adolescent resettled refugees’ experiences in Canada and Australia during the pandemic, as well as other resettled refugee populations in the U.S, such as Rohingya refugees. |
| **Reviewer 5** | | | |
|  | The manuscript title: “We will need to build up the atmosphere of trust again”: Perceptions of COVID-19 impacts on resettled refugee adolescents and educational and support services in the United States., Overall, this manuscript is quite well written., It appropriate to publish to journal. Please recheck all references, table and figure again. | Thank you. We have checked all references and tables. |  |
| **Reviewer 6** | | | |
|  | “We will need to build up the atmosphere of trust again”: Perceptions of COVID-19 impacts on resettled refugee adolescents and educational and support services in the United States. Overall concise, interesting and well written. Please recheck all references styles. | Thank you. We have rechecked all reference styles. |  |
| **Reviewer 7** | | | |
|  | The study is an opinion article about perceptions of interviewees, rather than a data driven quantitative study to assesses the outcomes. | This study is a qualitative study and we have taken efforts throughout the manuscript to emphasize this, as well as clearly indicate that this reflects service providers’ perspectives. As such, we have changed the title (see response to comment 2, below).  There are a range of epistemologies to which scientists ascribe, from the positive approach (which may necessitate a quantitative approach) to constructivist approaches. We argue that all these approaches have merit and can expand understanding of the constructs we seek to study. A constructivist epistemology is appropriate where it matches the research objectives and employs rigorous and transparent research methodology, as we believe is the case with this study. This study is grounded in a constructivist approach that values the knowledge of the experts working with refugee youth daily, and distills the common themes that arise from their experiences and understandings. |  |
|  | The title is an opinion expression rather than indicator of study’s key features. Authors should consider revising the title itself. | “We will need to build up the atmosphere of trust again”: Service providers’ perceptions of experiences of COVID-19 amongst resettled refugee adolescents” |  |
|  | The study has not factored in the dichotomy created by inputs from heterogenous populations of youth and caregivers, which may confound any conclusions driven. | The data in this study are based only on service providers’ reports. While they may be discussing heterogenous populations of youth and caregivers, we do not believe that we can adequately factor in the dichotomy that the reviewer highlights as the service providers did not indicate the exact demographics of the populations with which they were working when we interview them. |  |
